# Purtscher-like retinopathy as the manifestation of adult Still’s disease

**DOI:** 10.3205/oc000257

**Published:** 2025-10-02

**Authors:** Maria Lourdes Castro de Oliveira Figueiroa, Ricardo Danilo Chagas Oliveira, Gustavo Luiz Behrens Pinto, Maria Carolina Moura Costa Campos, Lizandra Almeida David da Silva Viana, Izabela Prado Viana, Mittermayer Barreto Santiago

**Affiliations:** 1 Escola Bahiana de Medicina e Saúde Pública, Salvador, Bahia, Brazil; 2 Division of Ophthalmology, Faculdade de Medicina da Universidade Federal da Bahia, Salvador, Bahia, Brazil; 3 Division of Rheumatology, Faculdade de Medicina da Universidade Federal da Bahia, Salvador, Bahia, Brazil; 4 Division of Gastroenterology, Escola Paulista de Medicina da Universidade Federal de São Paulo, São Paulo, Brazil

**Keywords:** adult-onset Still’s disease, Purtscher-like retinopathy, ocular involvement, infliximab

## Abstract

Adult Still’s disease (ASD) is a rare systemic inflammatory disorder for which ocular manifestations have rarely been described. We report a case of 38-year-old Brazilian woman with Purtscher-like retinopathy as a manifestation of ASD. She was diagnosed with Purtscher-like retinopathy based on fundoscopic findings, which revealed vasculitis with diffuse and bilateral perimacular cotton-wool exudates. She also presented with fever, arthritis, weight loss, and a diffuse cutaneous rash. Considering the severity of the retinopathy, she was initially treated with methylprednisolone pulse therapy and oral methotrexate, and later with infliximab, with the treatment changing due to partial recovery of vision loss. Purtscher-like retinopathy is a poor prognostic factor for ASD, as it can lead to permanent visual damage. Thus, early and aggressive immunosuppressive therapy is mandatory.

## Introduction

Purtscher’s retinopathy is a rare condition first described in 1910 in a patient with reduced post-traumatic visual acuity who exhibited hemorrhages and retinal clearing [1]. Although primarily associated with trauma, some systemic conditions can lead to a similar retinopathy, known as Purtscher-like retinopathy [2]. Among these conditions, acute pancreatitis, renal disease, thrombotic microangiopathies, cholesterol emboli, cryoglobulinemia, and diffuse connective tissue diseases (DCTDs), specifically systemic lupus erythematosus, systemic sclerosis, and dermatomyositis, stand out [2], [3].

## Case description

**Figure 1:**
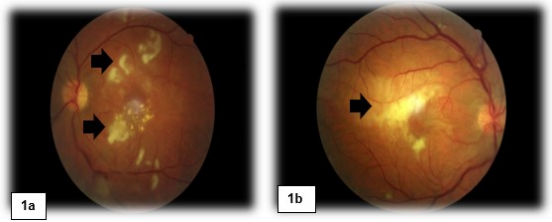
a: Fundoscopy of the right eye revealed papilla with physiological cupping and presence of peripapillary shunts, diffuse arteriolar attenuation associated venous engorgement, retinal hemorrhages in the four quadrants, exudates in the macular region. b: Fundoscopy of the left eye revealed papilla with clear physiological cupping and presence of peripapillary shunts, diffuse arteriolar attenuation associated venous engorgement, retinal hemorrhages in the four quadrants, exudates in the macular region.

**Figure 2:**
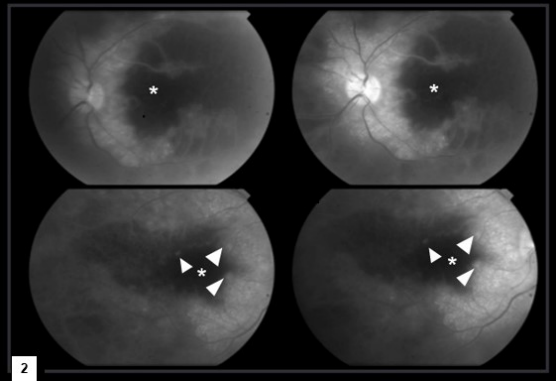
Sequence of angiography images in the late phase showed delayed circulatory times with slower filling of vessels in all phases of the angiogram, areas of non-perfusion (*), and late leakage (arrowhead).

Here, we present an exceptional case of Purtscher-like retinopathy with severe visual impairment as a manifestation of adult Still’s disease (ASD). A 38-year-old Brazilian woman, who was otherwise previously healthy, was admitted to our hospital complaining of intermittent fever, odynophagia associated with a weight loss of 15 kg, and polyarthritis of the hands, wrists, and knees for the last five months. During this period, she also developed a rapid progressive reduction of visual acuity bilaterally and reported recurrent chronic urticaria three months ago, with disseminated erythematous lesions refractory to the use of antihistamines and oral corticosteroids. She denied alcoholism, smoking, or illicit drug use. She had no previous hospitalization or family history of inflammatory disease. On physical examination, she had evanescent skin erythematous plaques on her trunk and limbs, in addition to tenderness in the joints of her hands, feet, and knees, albeit without swelling. Mild splenomegaly was also noted. No palpable hepatomegaly or adenomegaly was observed. Laboratory workup showed mild anemia without hemolysis pattern, neutrophilic leukocytosis, and normal platelet count. C-reactive protein and ferritin levels were elevated. Renal, hepatic, and pancreatic functions were normal. Autoantibodies and infectious tests were negative, as shown in Table 1 (Tab. 1). Additional evaluation with a tomographic study of the chest and abdomen revealed no abnormalities. Ophthalmologic examination showed visual acuity of counting fingers at 30 cm in both eyes. External examination revealed orthotropic eyes with full extraocular movements and intact motor reflexes. Anterior segment evaluation showed quiet eyes with clear corneas, well-formed and deep anterior chambers, iris without abnormalities, and a phakic lens. There were no signs of anterior chamber inflammation, and the vitreous was clearly visible. Funduscopic examination revealed vasculitis with diffuse bilateral perimacular cotton-wool exudate (Figure 1 (Fig. 1)). A complementary study with fluorescein angiography revealed delayed circulatory times in all phases of the angiogram, early hypofluorescence, slower filling of vessels, and late leakage, similar to the symptoms of Purtscher-like retinopathy (Figure 2 (Fig. 2)). 

Because other systemic disorders were excluded, the patient was diagnosed with ASD according to Yamaguchi’s criteria. Pulse therapy was initiated with 1 g methylprednisolone for three days, followed by 1 mg/kg of prednisone daily and oral methotrexate (15 mg/week) with gradual remission of systemic inflammatory activity. In addition, endolaser photocoagulation was performed. Ophthalmological reassessment one month later showed a slight reduction in diffuse and perimacular cotton-wool exudates. Immunobiological treatment was thus initiated with infliximab intravenously administered at 3 mg/kg, at weeks zero, two, six, and every eight weeks thereafter, with the treatment changing due to partial recovery of her vision. At the last evaluation, after 25 months of follow-up, the patient’s best-corrected visual acuity was 20/200 in both eyes.

## Discussion

Ocular involvement in ASD occurs in approximately 5% of patients, mainly in those with a severe systemic presentation [4]. The main manifestations are sicca syndrome, uveitis, episcleritis, inflammatory orbital pseudotumor, ptosis, diplopia, and nystagmus [5], [6]. Purtscher-like retinopathy associated with ASD is a rare condition [6]. In medical literature, we found only nine additional cases, consisting of five men and four women with a mean age of 33 years, most of whom had early ocular involvement and conditions associated with thrombotic microangiopathy (TMA) [4], as shown in Table 2 (Tab. 2). In the present case, the laboratory data and physical examinations did not fulfill the TMA criteria. Buyukavsar et al. also reported Purtscher-like retinopathy without TMA in a patient with ASD who was successfully treated with a high dose of oral prednisolone [7]. Therefore, in cases where Purtscher-like retinopathy is manifested early, as in our case, systemic immunosuppressive therapy may prevent the onset of TMA [8].

Purtscher’s retinopathy may present with visual field loss in the form of central, paracentral, or arcuate scotoma, with preservation of peripheral vision [1]. It usually occurs 24–48 hours after head or thoracic trauma, long bone fracture, or during systemic conditions such as acute pancreatitis, renal dysfunction, TMA, and DCTDs [2], [3], [9].

Its pathogenesis has not yet been well-defined [3]. Some evidence supports the idea that in addition to complement deposition, occlusion of precapillary arterioles by fibrin aggregates, leukocytes, and platelets leads to intravascular coagulation and focal ischemia [2], [3]. Particularly, in inflammatory diseases, the initial occlusive phenomenon may be related to endothelial dysfunction [2], [3].

The diagnosis of Purtscher’s retinopathy is based on ophthalmoscopic findings, such as “flaming” and diffuse retinal hemorrhages, and perimacular cotton wool nodules [3]. Purtscher’s flecken pathognomonic lesions are described in 50% of the cases [3]. It consists of multiple discrete areas of retinal whitening in the superficial aspect of the inner retina between the arterioles and venules [3]. These fundoscopic abnormalities are confined to the posterior pole, specifically within the macula [3]. Fluorescein angiography is an additional test that may reveal arteriolar occlusions with areas of hypofluorescence [3]. 

Currently, there is no well-established treatment for Purtscher-like retinopathy [3]. High-dose corticosteroids have variable efficacies and may improve visual outcomes in some patients [3]. In general, emphasis is placed on the treatment of underlying diseases [3]. However, most cases of Purtscher-like retinopathy show spontaneous remission [3]. Acute lesions tend to disappear within 1–3 months, even without treatment, and are replaced by atrophy of the pigment epithelium, pallor of the optic disc, and attenuation of retinal vessels [3]. However, when Purtscher-like retinopathy is associated with DCTD or TMA, it usually has a worse prognosis, with only partial recovery observed in approximately 70% of cases [10]. 

The treatment of ASD in an acute setting consists of nonsteroidal anti-inflammatory drugs and corticosteroids [11]. After achieving clinical and biochemical remission, gradual tapering of the corticosteroids is initiated. Blockade of methotrexate, Interleukin-1 (IL-1), and Interleukin-6 (IL-6) is reserved for relapsing and chronic disease [10]. In all cases described, corticosteroids were used, and in severe cases, additional immunosuppression was performed with blockade of methotrexate, IL-1, or IL-6 [11]. Most patients presented with partial recovery [11]. We used pulse therapy with corticosteroids followed by maintenance of oral steroid therapy and oral methotrexate, but it was ineffective for the recovery of the patient’s vision. Given the persistence of bilateral posterior uveitis despite the control of systemic inflammation, we chose tumor necrosis factor inhibitor (anti-TNF) therapy because it has already proven to be beneficial in non-infectious uveitis [11], [12]. Although we do not know the long-term therapeutic response, the patient showed partial improvement in visual damage. 

## Conclusion

Purtscher-like retinopathy is a poor prognostic factor for ASD, as it can lead to permanent visual damage. Therefore, early and aggressive immunosuppressive therapy is mandatory. 

## Notes

### Ethics statement and informed consent

The case report is a minimal risk study which was conducted in full compliance with the principles of the Declaration of Helsinki and Good Clinical Practice. All identifying patient information was kept confidential. Informed consent was obtained from the patient.

### Competing interests

The authors declare that they have no competing interests.

## Figures and Tables

**Table 1:**
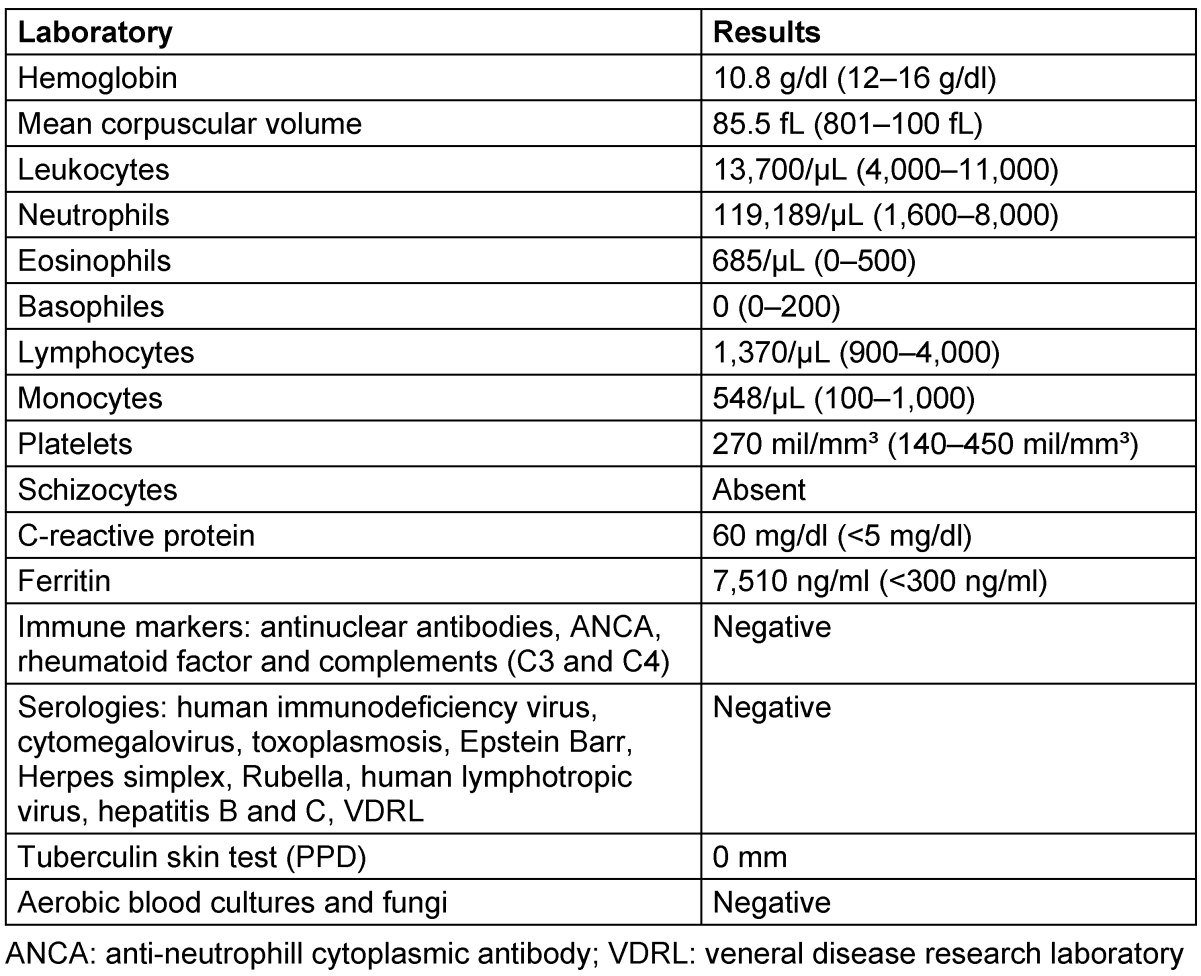
Main laboratory findings

**Table 2:**
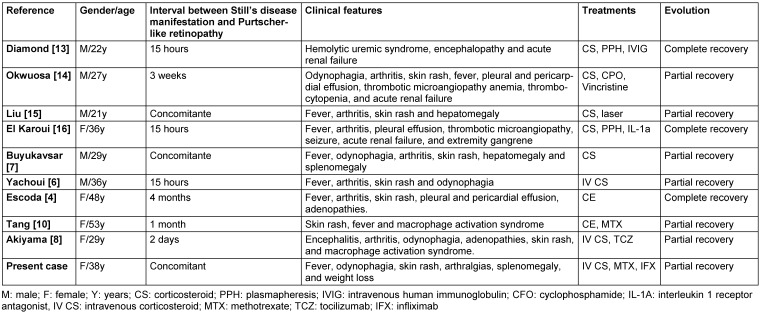
Clinical profile of patients with Purtscher-like retinopathy as a manifestation of adult Still’s disease
